# Purcell enhancement and polarization control of single-photon emitters in monolayer WSe_2_ using dielectric nanoantennas

**DOI:** 10.1515/nanoph-2022-0628

**Published:** 2023-01-16

**Authors:** Shaimaa I. Azzam, Kamyar Parto, Galan Moody

**Affiliations:** Electrical and Computer Engineering Department, University of California, Santa Barbara, CA 93106, USA; California Nanosystems Institute, University of California, Santa Barbara, CA 93106, USA

**Keywords:** dielectric nanoantennas, photonic integrated circuits, Purcell enhancement, quantum emitters, single photon sources, two-dimensional materials

## Abstract

Two-dimensional (2D) materials have shown great promise as hosts for high-purity deterministic single-photon sources. In the last few years, the underlying physics of single photon emission in 2D materials have been uncovered, and their optical properties have been improved to meet criteria for a variety of quantum technologies and applications. In this work, we take advantage of the unique characteristics of dielectric nanoantennas in manipulating the electromagnetic response on a sub-wavelength scale to localize and control defect-based single-photon emitters (SPEs) in 2D layered materials. We show that dielectric nanoantennas are capable of inducing high Purcell enhancement >20 and therefore brighter single-photon emission, which is characterized by a reduction of the emitters’ radiative lifetimes and enhancement of their brightness by more than an order of magnitude. We demonstrate that the sub-wavelength-scale dielectric nanoantennas can be designed to also impose a predetermined strain profile that determines the confinement potential of the SPE, leading to robust control over the optical polarization with up to 94% extinction ratio. The combination of large Purcell enhancement, polarization orientation, and site control through strain engineering demonstrates the advantages and unique capabilities of dielectric nanoantennas for enhancing the quantum optical properties of 2D SPEs for quantum information technologies.

## 1 Introduction

Since the initial discovery of single-photon emission and demonstration of photon anti-bunching with sodium atoms [1], significant research efforts have been focused on finding and optimizing an ideal single-photon source due to the great implications it would have on numerous quantum technologies, such as quantum cryptography, quantum communication, and quantum information processing [2–4]. Amongst the several platforms for which single photon emission has been reported, two-dimensional (2D) materials such as transition metal dichalcogenides (TMDs) have shown great promise as hosts for high-purity single-photon emitters (SPEs) with an overall performance comparable to established solid-state hosts [5–9]. Unlike SPEs in bulk solid-state materials [10, 11], the integration of 2D materials with photonic integrated circuits (PICs), such as foundry-processed silicon and silicon nitride PICs [12–16], is more straightforward. Also, due to their atomic thickness, light extraction from 2D materials is not hindered by the high refractive index of the host material, enabling theoretically high extraction efficiency close to unity. Recent reports have shown that the formation of the SPEs can be engineered in 2D flakes using strain [17, 18] or particle irradiation [19–22] to produce deterministic and highly ordered arrays of SPEs with electrically controllable switching of emission from the SPEs [23].

It is currently well established that the stable and scalable deployment of SPEs will require their integration with resonant structures to boost their emission rates as well as their extraction efficiencies [24]. The coupling with a well-defined cavity mode can increase the spontaneous emission rate through the Purcell effect and has therefore been a subject of extensive effort in SPEs in 2D materials and other material platforms. In addition, the lifetime shortening associated with a Purcell-enhanced photon emission can lead to increased indistinguishability between consecutively emitted photons [11, 25, 26]. Single photon emitters in 2D materials have thus far been integrated with various cavity geometries including plasmonic structures [27–30], dielectric on-chip cavities [31, 32], photonic crystals [33, 34], and microring resonators [16]. Plasmonic structures coupled to 2D materials have shown great advantages in forming strain-induced SPEs with high polarization purity [30] as well as significant Purcell enhancement due to their ability to confine the electromagnetic field into sub-wavelength regions [27, 30]. Despite their numerous advantages, plasmonic cavities are inherently lossy due to the use of metals, and extra materials are needed to shield the emitters from the metal leading to increased fabrication complexity and reduced emitter-hotspot overlap. Additionally, the most common plasmonic materials, such as gold and silver, are generally not compatible with standard CMOS fabrication processes, which hinders the on-chip large-scale deployment of 2D SPEs enhanced and engineered using plasmonic cavities. Therefore, all-dielectric structures are preferred due to the lack of material losses. Amongst the various cavity configurations studied thus far [28, 31, 32, 34], dielectric nanoantennas are promising due to their ultra-compact footprint, their ability to integrate with on-chip all-photonic circuitry, and their ability to engineer their response [35–37]. High refractive index nanoantennas are able to support strong electric and magnetic resonances at the nanoscale, which has proven very powerful for engineering light–matter interaction for a variety of applications.

**Figure 1:**
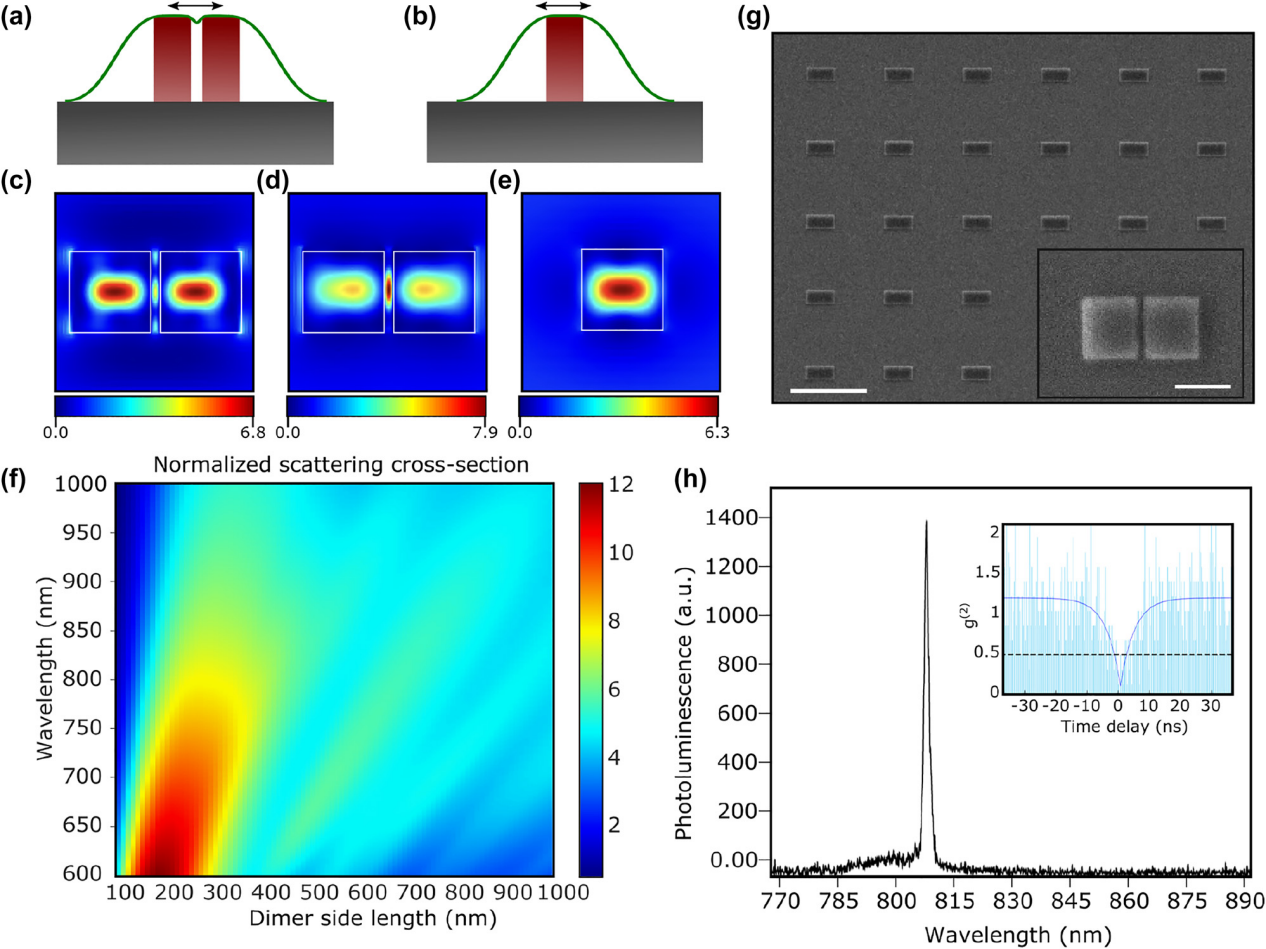
Engineering single-photon emission using dielectric nanoantennas. A schematic of a 2D flake on top of a (a) dimer and (b) monomer nanoantenna. The scattered electric field normalized to the incident wave’s amplitude ${\left(\left|E_{\text{s}}|/|E_{0}\right|\right)}^{2}$ shows the field enhancement of (c) a square-shaped dimer with a side length of 475 nm and a gap width of 40 nm at a wavelength 700 nm, (d) similarly for a wavelength 800 nm, and (e) of a single square-shaped nanoantenna with a side length of 475 nm at 800 nm wavelength. (f) Simulated scattering cross-section of the square dimer with varying side length. The scattering cross-section is normalized to the physical area of the dimer. (g) A scanning electron microscope image of the square-shaped dimer with a side length of 475 nm and a gap width of 40 nm. The scale bar is 5 μm. Inset is a zoomed-in scanning electron microscope image of the cross-section of a single dimer to show the gap. The scale bar is 500 nm. (h) Photoluminescence spectrum of a representative SPE in monolayer WSe_2_ on top of a dimer measured at 5 K. The inset shows the second-order auto-correlation measurement demonstrating $g^{\left(2\right)}\left(0\right)=0.11$ . The data are raw with no correction for the background or detector dark counts.

In this work, we integrated 2D SPEs with silicon nitride nanoantennas to localize and control the single photon emission. We show that our choice of a square-cross-sectional dimer nanoantenna is a unique configuration for strain-induced SPEs as it can host electromagnetic hot spots down to a few tens of nanometers, leading to photoluminescence enhancement due to the Purcell effect by more than an order of magnitude (Figure 1). In addition, we show that the strain profile imposed by the geometry of dimer nanoantennas can be selected to give rise to a specific polarization of the SPEs with an extinction ratio up to 94%. The results demonstrated here provide evidence that dielectric nanoantennas are a great platform for strain-induced SPEs that simultaneously enables site control, polarization control, and deterministic bright single photon emission. While the lifetime, yield, and polarization of the single photons on 2D materials have been explored here, nanoantennas can be used to alter other radiation properties of the emitted SPE, such as the angle of radiation and the scattering efficiency [38, 39].

## 2 Results and discussion

We focus on the engineering of the brightness and polarization of SPEs hosted in monolayer tungsten diselenide (WSe_2_) using sub-wavelength all-dielectric nanoantenna structures. Nanoantennas with square cross-sections are chosen due to their ability to not only provide Purcell enhancement for the SPEs, but also their potential to engineer the strain profile, which in turn controls the SPE polarization [40]. Compared to a monomer nanoantenna such as a square or cylindrical pillar, a dimer nanoantenna comprising a pair of antennas with a sub-wavelength gap between them can boost the field enhancement due to the gap mode, which has been the source of numerous nonlinear light–matter enhancements [35, 36].

A schematic of the dimer and the single square cross-sectional nanoantennas is shown in Figure 1(a) and (b), respectively. The normalized electric field calculated at 5 nm on top of the dimer at a wavelength of 700 nm and 800 nm, and of the single nanoantenna at wavelength 800 nm, are shown in Figure 1(c)–(e), respectively. The field enhancement is calculated as the relative electric field intensity *E*_s_ scattered upward on the nanoantenna (5 nm above) normalized to the amplitude of the incident plane wave $E_{0}\;{\left(\left|E_{\text{s}}|/|E_{0}\right|\right)}^{2}$ . The maximum field enhancement in the single nanoantenna is limited to the center of the structure, as shown in Figure 1(e), which does not align with the points of maximum strain where the SPEs tend to appear at the edges or corners of the structure [21]. For the dimers, significant field enhancement can be observed at the edges and the corners, which correspond to the locations of strained monolayers where SPEs tend to appear. In addition to the field enhancement and the Purcell enhancement, nanoantenna structures can be used to directionally scatter single photons with high efficiency, analogous to a normal antenna. Simulation results of the normalized scattering cross-section of the dimer structures as a function of the dimer side length are shown in Figure 1(f). The scattering cross-section is simulated through illuminating the structure from the top (air side) with a plane wave. It is worth mentioning that the simulations are carried out for each individual dimer without any periodicity. The calculated scattering cross-section is then normalized to the physical size of the dimer to extract the normalized scattering cross-section. More than an order-of-magnitude enhancement of the scattering cross-section relative to the physical cross-section of the dimer can be obtained. While boosting the scattering cross-section alone does not guarantee a better collection efficiency of the emitted light, optimization of the nanoantenna’s radiation patterns can be used to specifically target enhanced directivity [41]. This is a vital point in boosting the collection efficiency of generated single photons to mitigate the loss of photons due to high-index photonic substrates.

We chose to use silicon nitride (SiN) for the nanoantennas in this work due to its broad spectral transparency window, high refractive index (∼2), and established processing techniques. A 500 nm thick film of low-stress SiN was deposited on a pre-cleaned quartz substrate using plasma-enhanced chemical vapor deposition (PECVD) followed by sputtering ∼30 nm of ruthenium that is used as a hard mask and electron dissipation layer [42]. Patterns are then written using electron beam lithography and etched using inductively coupled plasma etching. A scanning electron microscope image of a fabricated dimer structure prior to the monolayer transfer is shown in Figure 1(g). Following photonic device fabrication, a monolayer WSe_2_ flake is transferred onto the sample using an all-dry transfer technique [43]. All measurements are carried out inside a closed-cycle cryostat where the sample is cooled down to ∼5 K. The sample is excited using a 660 nm pulsed laser with a variable repetition rate. The photoluminescence spectrum is recorded using a spectrometer and silicon CCD. Laser light is focused on the sample through a long-working distance objective with 0.55 numerical aperture, resulting in a ∼1.37 μm focused spot on the sample. The laser spot is scanned across the sample for initial imaging of the SPEs. Nearly all dimers covered by a monolayer WSe_2_ flake exhibited at least one sharp emission peak corresponding to SPE. The majority of the measure SPEs was well-isolated spectrally, with a representative spectrum shown in Figure 1(h). The second-order auto-correlation measurement demonstrating a pronounced photon antibunching behavior with $g_{2}^{\left(0\right)}$ = 0.11 is shown in the inset. The auto-correlation data are shown with no correction for the background or detector dark counts.

**Figure 2:**
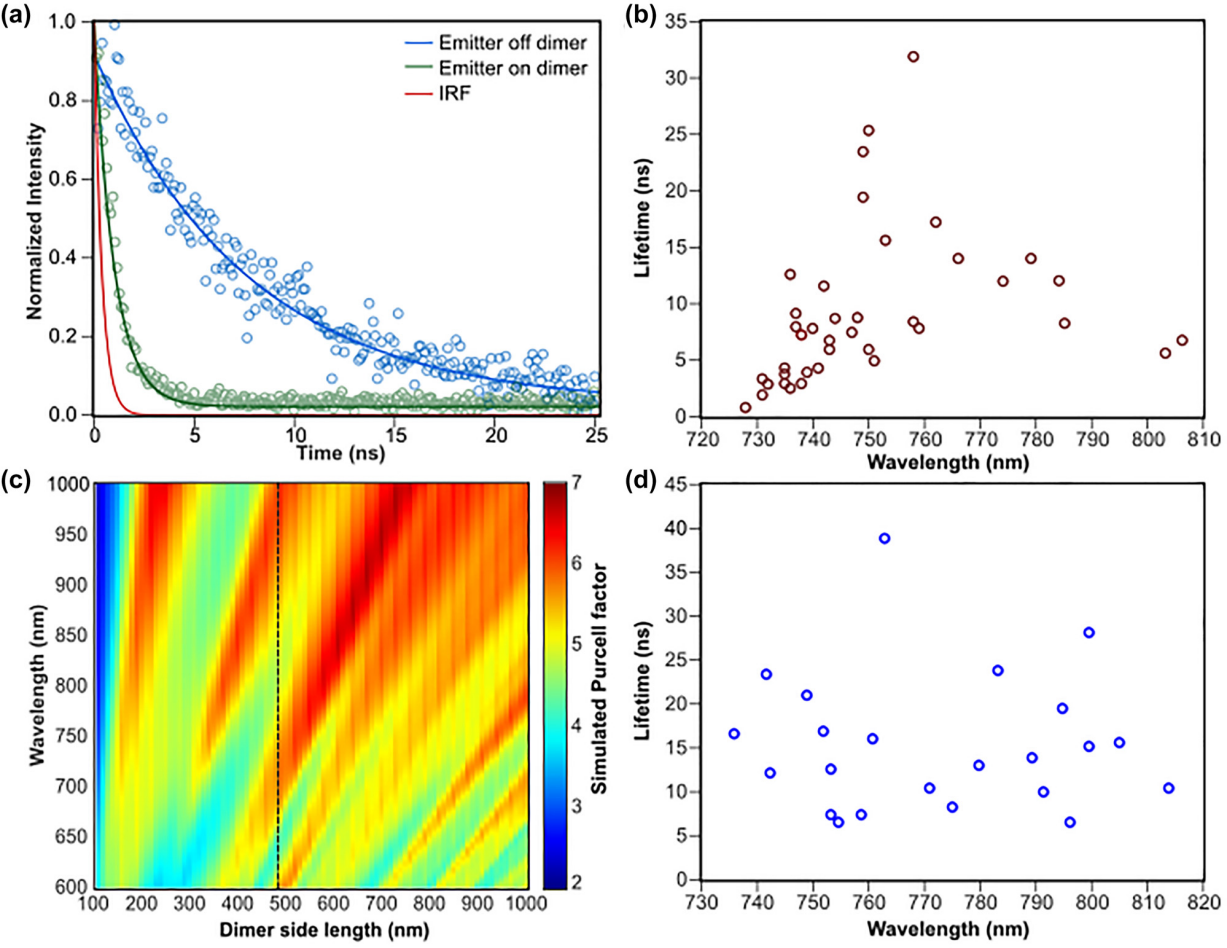
Purcell enhancement of single photon emitters on a dimer nanoantenna. (a) The lifetime of a single photon emitter on a dimer (green dots), a random emitter off-dimer (blue dots), and the instrument response function (IRF, red). The solid lines are the exponential fits resulting in 0.49 ns (corrected for the instrument response) and 7.3 ns for emitters on and off dimers, respectively. (b) A scatter plot of the lifetimes of SPEs from the dimer sample as a function of their wavelength. (c) Simulated Purcell factor of an emitter positioned 5 nm on top of the center of a square dimer perpendicular to the dimer’s gap with a 40 nm gap as a function of the dimer side length. The black dashed line represents the fabricated sample with a side length of 475 nm with which the measurements are performed. (d) A scatter plot of the lifetimes of a group of random emitters.

Time-resolved lifetime measurements are performed for the majority of the studied SPEs using a pulsed 660 nm laser at a 40 MHz repetition rate. Here, we make a distinction between two types of SPEs: those that are formed due to the intentional strain induced by the dimers (dimer SPEs), and another group of SPEs randomly appearing in flakes directly transferred on the quartz substrate (random SPEs). The random SPEs can be attributed to unintentional pockets of strain induced during the transfer process, bubbles between the substrate and flake, or breakage and folds in the monolayer [44]. Lifetimes of the random SPEs exhibited a wide spread emitter lifetimes spanning 6 ns to 40 ns with the average being 11.25 ns Figure 2(d). A representative lifetime of a random SPE is shown as the blue dots in Figure 2(a) and is fitted to the blue line with a lifetime of 7.3 ns.

A sample of the lifetime of emitters formed due to the strain induced by the dimers with a side length of 475 nm is shown in Figure 2(b) as a function of the emitter’s wavelength. While the measured lifetimes of on-dimer emitters span a wide range, this is to be expected. Two key differences have been observed between on- and off-dimer SPEs: (1) the minimum lifetime in the dimer-SPEs is found to be much shorter than those of random ones, and emitters with sub-ns lifetimes were measured as shown by the green dots in Figure 2(a) demonstrating an SPE with 0.49 ns lifetime, corrected for the instrument response function (IRF); and (2) unlike the random emitters’ lifetimes, the short lifetimes (<5 ns) of the dimer-SPEs are clustered in the 730–745 nm wavelength range, which is in agreement with the expectation of the largest Purcell enhancement shown in the simulations in Figure 2(c). As evident from Figure 2(c), in which the Purcell factor is calculated as a function of the dimer side length assuming a dipole source oriented across the gap, as drawn in Figure 1(a), the Purcell enhancement is wavelength dependent. For a dimer side length of 475 nm, highlighted by the dashed line in Figure 2(c), the maximum Purcell factor lies between wavelengths 670 nm and 740 nm. SPEs in WSe_2_ appear below the exciton energy, which corresponds to ∼725 nm and therefore, according to the prediction from the simulation in Figure 2(c), emitters in the range 725 nm–740 nm should have the shortest lifetimes, which is indeed the case as shown in Figure 2(b). This serves as additional evidence that the shortening of the lifetimes observed on the emitters found on top of dimers is indeed due to the Purcell effect. While controlling the wavelength of the SPEs in 2D materials has been elusive, the results shown here present a promising step to cluster all bright, Purcell-enhanced emitters into a tight wavelength spread using the dielectric nanoantennas. By comparing the average (shortest) measured lifetime for emitters between 725 and 735 nm on dimers to the average lifetime of random emitters off dimers, we find a Purcell enhancement of 4.1 (22). Since the Purcell-enhanced emitters are formed primarily due to strain exerted by the nanoantennas, we rely on a comparison of lifetime measurements for coupled and uncoupled emitters, because correlations cannot be drawn from measurements of the intensity for a given emitter. Due to that, our way of calculating the Purcell enhancement might not reflect that experienced by each individual emitter. This could explain the difference between the predicted Purcell factor from the simulation and that measured experimentally. Another factor that might play a role in such discrepancy is fabrication process variations. The nominal gap size in this work is 40 nm in the dimers, which pushes the limit of our nanofabrication capabilities. Variation in the gap sizes can lead to smaller gaps, which would exhibit Purcell enhancement compared to what is shown in Figure 2(c). Moreover, due to the very small gap size, some gaps are not etched all the way through to the substrate, which can alter the field confinement profile and potentially increase the Purcell factor in the experiments.

Prior studies have demonstrated that SPEs in TMDs are linearly polarized; however, the orientation of the polarization is random, which hinders the integration with resonant structures. Very recently, the manipulation of the polarization orientation of TMD SPEs by engineering their confinement potential through deformation strain induced by pre-patterned substrates has been successful in the control of single photon polarization [40, 45]; however, using these schemes to integrate an SPE with a cavity can still be a tedious task due to the high-resolution alignment and the compatibility considerations of different platforms and materials. This was the driving force behind our choice of the geometry of the dimer with a square cross-sectional area as opposed to the common choice of circular cross-sections. The linear edges of the dimers promote the formation of an asymmetric confinement potential leading to quasi one-dimensional excitons [45] that are self-aligned to the cavity formed by the high refractive index dimer. It has been shown that for such quasi one-dimensional excitons, fine-structure-split states at higher energy are effectively suppressed. This leads to suppression of the optical oscillator in the perpendicular direction while favoring the excitonic dipole emission along the edge of the confinement potential [45].

**Figure 3:**
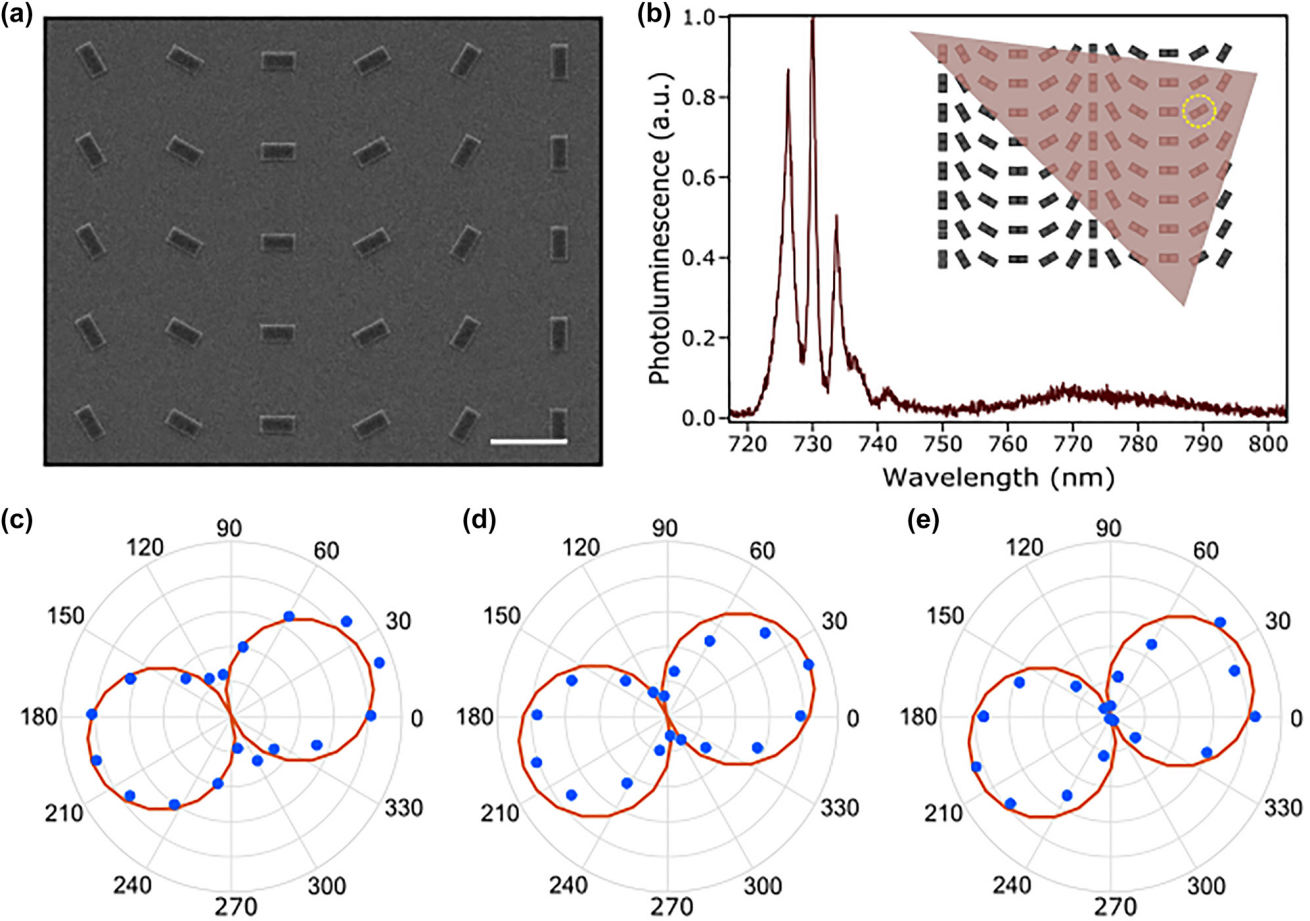
Polarization control of single photon emitters using dielectric nanoantennas. (a) A scanning electron microscope image of a dimer sample with varying the axis of the dimer by step of 30*°* and a side length of 475 nm and a 40 nm gap. The scale bar is 5 μm. (b) Photoluminescence spectrum of SPEs on a dimer showing three single-photon emitters at a dimer oriented at 30*°* angle as highlighted by the yellow circle in the schematic inside the inset. The inset shows a schematic of the dimer sample fabricated and measured with a monolayer WSe_2_ transferred onto the sample. (c)–(e) Polarization measurements of the three emitters shown in (b) showing an angle of ∼30° matching that of the dimer orientation.

Figure 3 summarizes the results of using the square cross-sectional dimers to manipulate the polarization orientation of SPEs. A sample with the same 475 nm side length was fabricated from SiN on quartz as previously described with each dimer rotated by an angle of 30° relative to its neighbor as can be seen in Figure 3(a). A photoluminescence spectrum of SPEs localized on a dimer is shown in Figure 3(b), which features three SPEs. A schematic of the sample is shown in the inset of 3(b) with the dimer being measured highlighted in the yellow dashed circle indicating a dimer tilted by a 30° angle with respect to the horizontal axis. The polarization of each of the SPEs in Figure 3(b) has been measured by rotating a half-wave plate in front of a linear polarizer in the photoluminescence collection path prior to the spectrometer, shown in Figure 3(c)–(e). We observe an excellent agreement between the polarization angle of all SPEs on the dimer and the dimer’s rotation angle, both being near 30°. It is important to note that due to the fact that the laser spot size is ∼1.37 μm, the entire dimer can be illuminated simultaneously. Thus, we occasionally observe that the SPE polarization and the dimer orientation do not always agree. Similar behavior was reported previously in larger patterns with >1 μm-scale lengths in which the individual edges were resolved and the polarization matched the structural orientation closely (but without a demonstrated Purcell enhancement) [45]. In our study, the yield of SPEs with the polarization angle matching those of the underlying dimer was 56%, which we believe will be enhanced drastically for a more tightly focused laser spot.

## 3 Conclusions

In summary, we have demonstrated that dimer nanoantennas with square cross-sections offer a unique platform for strain-induced single photons in 2D materials. Such a platform offers the ability to localize high purity (∼90%) SPEs through strain, enhances the radiative emission rates through the Purcell factor by more than a factor of 20, and controls the SPE polarization through strain engineering. SPEs with controllable polarization and a high extinction ratio of up to 94% have been demonstrated. Moreover, simulations have shown that nanoantennas can be utilized to achieve a scattering cross-section larger than the SPE physical size by more than an order of magnitude, which can be used to enhance the scattering and collection of the generated single photons. In general, dielectric nanoantennas, especially square dimers, can be exploited to simultaneously engineer several properties of strain-induced single photon emitters in 2D materials, opening up new opportunities for quantum information applications.
